# Application Research on the Lightweight Design and Optimization of Carbon Fiber Reinforced Polymers (CFRP) Floor for Automobile

**DOI:** 10.3390/polym14214768

**Published:** 2022-11-07

**Authors:** Shuai Zhang, Hao Song, Liyou Xu, Kefang Cai

**Affiliations:** 1 College of Vehicle and Traffic Engineering, Henan University of Science and Technology, Luoyang 471003, China; 2 Z-One Technology Co., Ltd., Shanghai 201800, China; 3 State Key Laboratory of Automotive Simulation and Control, Jilin University, Changchun 130022, China

**Keywords:** carbon fiber reinforced polymers (CFRP), automobile floor, material performance test, integrated structural design, structure layer design

## Abstract

In order to improve the lightweight level of the automotive floor, reduce material application cost, and improve integrated process manufacturing performance through structural design and optimization, this article proposes a design method to link conceptual design and detailed design and optimize the composite floor by combining free size optimization and size optimization methods. The basic theory of composite mechanics is expounded from the stress-strain theory of single-layer plates, and the stiffness and strength theory of laminated plates, which provides theoretical support for the structural design, material design, and allowable value design of composites. The mechanical properties of CFRP were tested to obtain the basic material parameters of CFRP T300/5208. With the material parameters, the CFRP floor super layers are established in Optistruct software. The shape of the floor super layers is optimized by using the free size optimization method, with the body-in-white (BIW) lightweight coefficient as the objective and the BIW performance as the constraints. The BIW lightweight coefficient is reduced from 4.35 to 4.20 after free size optimization, and the layer blocks shape is obtained and clipped based on engineering application. With the floor mass as the objective and the BIW performance as the constraints, the size optimization of the floor layer blocks thickness is optimized. Then the number of floor layers is obtained, and the CFRP floor is established in Fibersim software. Use the simulation analysis method to compare and verify the performance of the floor before and after optimization. The results show that the failure index of the floor is far less than the failure standard, while the mass of the CFRP floor is reduced by 6.8 kg compared with the original steel floor, which an improvement rate reaching 27.5%. The design and optimization methods presented in this article provide a reference for the design and application of the CFRP floor.

## 1. Introduction

Lightweight materials are an important means to achieving energy saving and an emission reduction in automobiles. For every 10% reduction in automobile curb mass, its fuel consumption can be reduced by 6–8%, and the corresponding exhaust emissions will be reduced by 4.5% [1,2,3]. The automobile floor is an important load-bearing assembly with complex stress conditions, and its lightweight design has an important impact on the static performance, noise, vibration, and harshness (NVH) performance, fatigue performance, and crash safety performance of the whole automobile [4,5,6].

With the continuous development of new materials such as high-strength steel, aluminum-magnesium alloy, and carbon fiber reinforced polymers (CFRP), the research of hybrid material automobiles has begun to receive more and more attention [7,8,9,10]. Compared with aluminum alloy and steel, CFRP can effectively reduce weight by 25–30% and 40–60%, its strength and stiffness are 5–7 times that of steel, and it has better corrosion resistance, fatigue resistance, and impact resistance [11,12]. The anisotropic mechanical property characteristics of composites are essentially different from those of metallic materials, and their design complexity and flexibility are more prominent, which have great potential in the lightweight design of automobiles [13,14].

The automobile floor not only bears the weight load of the occupants directly, but also bears the road impact load transmitted by the tires through the suspension and is the main body of the automobile to bear the load [15,16,17]. The floor with high strength and stiffness can better improve the low-order modal frequency of the body and improve the NVH performance and handling stability of the automobile. During the collision, it can better transmit and attenuate the collision energy and reduce the injury index to the occupants [18,19,20]. Therefore, the floor structure should not only have the role of bearing the combined load of tensile, compression, bending, and shear, but also have a certain strength and stiffness to ensure the performance of the automobile. On the premise of meeting the use requirements, how to select the raw materials of composite floors and reduce the manufacturing cost are the key issues in the design and development of composite floors.

CFRP has high strength and stiffness, which can effectively reduce the weight of structural components and improve crash safety in automobile design. Liu and Guan studied composite carriage floor plywood with different structures and evaluated the mechanical properties, thermal and sound insulation properties of the composite plywood [21]. Sukmaji et al. studied the application of sandwich polypropylene honeycomb core with carbon/glass fiber composite skin (SHCG) as the matrix material in electric car floor components and performed finite element analysis of SHCG based electric car floor component materials [22]. Carrera et al. reduced its weight by 50% and the number of components by 70% under the condition of meeting the existing requirements of the torsional and bending stiffness of steel floor while evaluating the crashworthiness of composite floor chassis design through side column crash test [23]. Tang et al. designed and produced a new CFRP floor structure for high-speed train carriages, providing an effective CFRP equipment design scheme with a weight reduction of about 35.7% compared to conventional metal structures [24]. Ji et al. used PAM-RTM to simulate the flow paths during vacuum assisted resin infusion molding of automotive front floors and performed variable compression molding tests on the floors, and the results of the research are of great significance for the application of CFRP [25].

In the aspect of composite layer design, Xu et al. classified and compared various optimization problems and methods in the design of composite laminated structures, expounded three types of problems of constant stiffness design, variable stiffness design, and topology optimization, with introduced optimization design methods such as gradient method, heuristic method, and hybrid method [26]. Lee et al. studied the manufacturing of CFRP side beams and the shape of single cap-shaped cross-sectional and analyzed the failure mode and energy absorption characteristics of members according to the stacking conditions such as fiber orientation angle and cross-sectional shape [27]. Hwang et al. studied the energy absorption characteristics of CFRP cap section members under the axial impact failure test and conducted an axial impact failure test on each section member [28]. Liu and Paavola proposed an optimization method based on the gradient projection algorithm, with used the interior point penalty function method to transform the lightweight design optimization model into a series of linear constraints optimization problems and used the proposed optimization method to perform the lightweight design of two composite laminates [29]. Liu used Euler-Bernoulli beam theory to deduce the analytical sensitivity of eigenvalues to fiber volume fraction and used the Taylor series to transform the optimization model into a linear programming problem for the lightweight design of composite laminated beams with different boundary conditions [30].

The lightweight structure design must comprehensively consider the balance of materials, technology, and structure. In this article, a lightweight automobile floor is designed by selecting the epoxy resin CFRP T300/5208, and the basic performance parameters of the composite are obtained through the mechanical property test of the composite. Based on the integrated design characteristics of composites, this article proposes a design method for CFRP floor that runs through the conceptual design, detailed design, and optimization design stages, which is of great significance to realize the integrated structural-material-process synergistic optimization design of floor.

## 2. Selection of Raw Materials for Composite Floor

### 2.1. Selection of Fiber Reinforcement Material

The properties of the reinforcement materials determine the mechanical properties of the composite material, and the fiber reinforcement materials are the main force point [31]. Automobile composite floor should not only have a high load-bearing capacity but also be able to withstand the combined effects of tensile, compressive, bending, shear, and other loads from all directions. Therefore, fiber reinforcement materials are required to have the characteristics of high strength and modulus. The overall strength of carbon fiber is higher than that of glass fiber and aramid fiber, and it has excellent resistance to high temperatures and corrosion. Aramid fiber with the increase in temperature, there will be a substantial reduction in strength, compression properties are poor, compression strength is relatively low, while glass fiber has the disadvantage of brittleness [32]. By comparing the performance parameters of typical reinforced fibers in Table 1, it can be seen that carbon fiber T300 has high specific strength and specific modulus, which is more conducive to lightweight design of the structure under cost control while ensuring strength and stiffness. Therefore, carbon fiber T300 is selected as the fiber continuous reinforcement material of composite floor structure in this article.

### 2.2. Selection of Composite Matrix

The matrix material, as an important part of the composite material, plays an important role in load transfer and distribution and, also, determines the interlaminar and transverse mechanical properties and shear properties of composite material, which has an important impact on the load-bearing capacity, fatigue resistance and impact resistance of structure [33,34,35,36].

Although the matrix material does not determine the mechanical properties of the composite material, the matrix material is an important factor in whether the fiber reinforcement material can maximize its performance, so the selection of the matrix material and the fiber reinforcement material should have a certain degree of suitability. At present, the commonly used resin matrix in automobile structure design mainly includes epoxy resin, vinyl resin, and polyester, among which epoxy resin is widely used because of its low curing shrinkage, good bonding, good toughness, and excellent processability [37,38,39]. Since automobile floor will be subjected to the impact load of the road and the reciprocating fatigue load during the load-bearing process, the matrix materials are required to have certain toughness and fatigue resistance, especially excellent interlaminar shear resistance. By comparing the performance parameters of typical thermosetting resins in Table 2, it is found that the epoxy resin has good comprehensive properties, especially the mechanical properties of the structure can be improved with small bending strain. Also, given that epoxy resin has good combination with reinforced carbon fibers, which is conducive to improving the interfacial properties of the composite [40]. Therefore, this article selects epoxy resin 5208 as the matrix material for composite floor structure design.

## 3. Basic Theory of Composite Mechanics

Composites have anisotropic elastic properties and are mainly used in structural design in the form of laminates. The mechanical analysis of anisotropic and isotropic materials has the same equilibrium equations, geometric equations, coordination equations, and boundary conditions, and the main difference is that the constitutive equations of stress and strain are different. When analyzing the mechanical properties of composites, the following conditions are often assumed to hold [41,42,43]: (1) Assume that the laminate is continuous; (2) Assumed that the unidirectional laminates are homogeneous; (3) Assumed that the unidirectional laminates are orthotropic anisotropic; (4) Assume that the laminates are linearly elastic; and (5) Assumed that the deformation of laminate is very small.

### 3.1. Constitutive Model of Composite Single-Layer Plate

**Figure 1:**
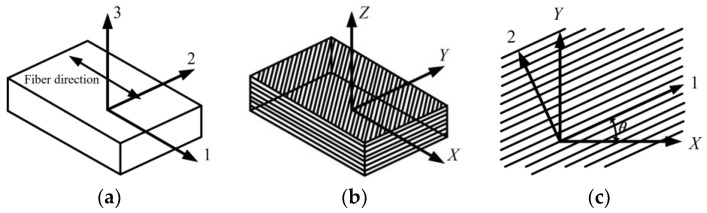
Material coordinate system of laminate: (**a**) Single-layer coordinate system; (**b**) Laminate coordinate system; (**c**) Positive rotation of shaft.

The fiber reinforced composite single-layer plates studied in this article are made of continuous and parallel anisotropic carbon fibers laid in the matrix. The fiber direction is specified as the first principal direction of the material, represented by 1, and the other two principal directions perpendicular to the fiber are represented by 2 and 3, respectively, as shown in Figure 1a. When analyzing the laminate, select the center surface equidistant from the upper and lower surfaces as the benchmark and, then, establish the reference coordinate system *XYZ*, as shown in Figure 1b. The angle between the direction of single-layer plate 1 and *X* direction is *θ*, which is defined as the layer angle of the single-layer plate in the laminate, and the direction is specified as positive when turning counterclockwise from the *X*-axis to the 1-axis, as shown in Figure 1c.

Since laminate is composed of a single-layer plate as the basic unit, the analysis of the strength and stiffness of a single-layer plate is the basis for the analysis of the strength and stiffness of the laminate. The plane thickness of the single-layer plate is very small compared with the other two directions, which can be considered as a plane stress-strain state *σ*_3_ = *τ*_23_ = *τ*_31_ = 0, so only *σ*_1_, *σ*_2_, *τ*_12_ and other in-plane stress components need to be considered [44].

For orthotropic anisotropic materials, the stress-strain relationship of single-layer plates is as follows:

(1) $$\left\{\begin{array}{l} \varepsilon_{1}\\ \varepsilon_{2}\\ \gamma_{12} \end{array}\right\}=\left[\begin{array}{ccc} S_{11} & S_{12} & 0\\ S_{12} & S_{22} & 0\\ 0 & 0 & S_{33} \end{array}\right]\left\{\begin{array}{l} \sigma_{1}\\ \sigma_{2}\\ \tau_{12} \end{array}\right\}=\left[S\right]\left\{\sigma \right\}$$

where *ε*_1_ and *ε*_2_ are the principal strains in directions 1 and 2, respectively; *γ*_12_ is the shear strain; *σ*_1_ and *σ*_2_ are the principal stresses in directions 1 and 2, respectively; *τ*_12_ is the in-plane shear stress; [*S*] is the flexibility matrix, which is used to represent the relationship within the unit; and [*Q*] is the reduced stiffness matrix, which is used to characterize the relationship between force and deformation of the unit body.

In the flexibility matrix [*S*], *S_ij_* is:

(2) $$S_{11}=\frac{1}{E_{1}},\;S_{22}=\frac{1}{E_{2}},\;S_{33}=\frac{1}{G_{12}},\;S_{12}=-\frac{\mu_{21}}{E_{1}}=-\frac{\mu_{12}}{E_{2}}$$

where *E*_1_ and *E*_2_ are the elastic moduli in the direction 1 and 2, respectively; *G*_12_ is the shear modulus; *μ*_21_ and *μ*_12_ are Poisson’s ratios in the direction 1 and 2, respectively.

By matrix transformation of Equation (1), the stress-strain relationship of single-layer plate can be obtained as follows:

(3) $$\left\{\begin{array}{l} \sigma_{1}\\ \sigma_{2}\\ \tau_{12} \end{array}\right\}=\left[\begin{array}{ccc} Q_{11} & Q_{12} & 0\\ Q_{12} & Q_{22} & 0\\ 0 & 0 & Q_{33} \end{array}\right]\left\{\begin{array}{l} \varepsilon_{1}\\ \varepsilon_{2}\\ \gamma_{12} \end{array}\right\}=\left[Q\right]\left\{\varepsilon \right\}$$

where [*Q*] is the reduced stiffness matrix.

The reduced stiffness matrix [*Q*] is obtained from the inverse of the two-dimensional flexibility matrix [*S*].

(4) $$Q_{11}=\frac{E_{1}}{1-\mu_{12}\mu_{21}},\;Q_{22}=\frac{E_{2}}{1-\mu_{12}\mu_{21}},\;Q_{33}=G_{12},\;Q_{12}=\frac{\mu_{12}E_{1}}{1-\mu_{12}\mu_{21}}=\frac{\mu_{21}E_{2}}{1-\mu_{12}\mu_{21}}$$

Since the material principal direction of the orthotropic anisotropic single-layer plate is inconsistent with the direction of the actual coordinate system, it is necessary to transform the stress-strain relationship from the 1-2 coordinate system to the *X*-*Y* coordinate system. The stress transformation equation of a single-layer plate is as follows:

(5) $$\left\{\begin{array}{l} \sigma_{1}\\ \sigma_{2}\\ \tau_{12} \end{array}\right\}=\left[\begin{array}{ccc} \cos^{2}\theta & \sin^{2}\theta & 2\sin\theta \cos\theta \\ \sin^{2}\theta & \cos^{2}\theta & -2\sin\theta \cos\theta \\ -\sin\theta \cos\theta & \sin\theta \cos\theta & \cos^{2}\theta -\sin^{2}\theta \end{array}\right]\left\{\begin{array}{l} \sigma_{x}\\ \sigma_{y}\\ \tau_{xy} \end{array}\right\}$$

The corresponding strain transformation equation is as follows:

(6) $$\left\{\begin{array}{l} \varepsilon_{1}\\ \varepsilon_{2}\\ \gamma_{12} \end{array}\right\}=\left[\begin{array}{ccc} \cos^{2}\theta & \sin^{2}\theta & \sin\theta \cos\theta \\ \sin^{2}\theta & \cos^{2}\theta & -\sin\theta \cos\theta \\ -2\sin\theta \cos\theta & 2\sin\theta \cos\theta & \cos^{2}\theta -\sin^{2}\theta \end{array}\right]\left\{\begin{array}{l} \varepsilon_{x}\\ \varepsilon_{y}\\ \gamma_{xy} \end{array}\right\}$$

Equations (5) and (6) can be simplified as follows:

(7) $$\left\{\begin{array}{l} \sigma_{x}\\ \sigma_{y}\\ \tau_{xy} \end{array}\right\}={\left[T_{\sigma }\right]}^{-1}\left\{\begin{array}{l} \sigma_{1}\\ \sigma_{2}\\ \tau_{12} \end{array}\right\},\;\left\{\begin{array}{l} \varepsilon_{x}\\ \varepsilon_{y}\\ \gamma_{xy} \end{array}\right\}={\left[T_{\varepsilon }\right]}^{-1}\left\{\begin{array}{l} \varepsilon_{1}\\ \varepsilon_{2}\\ \gamma_{12} \end{array}\right\}$$

where [*T_σ_*]^−1^ and [*T_ε_*]^−1^ are the inverse matrices of [*T_σ_*] and [*T_ε_*], respectively.

Combining Equations (2) and (6) yields Equation (8) as follows:

(8) $$\left\{\begin{array}{l} \sigma_{x}\\ \sigma_{y}\\ \tau_{xy} \end{array}\right\}={\left[T_{\sigma }\right]}^{-1}\left\{\begin{array}{l} \sigma_{1}\\ \sigma_{2}\\ \tau_{12} \end{array}\right\}={\left[T_{\sigma }\right]}^{-1}\left[Q\right]\left[T_{\varepsilon }\right]\left\{\begin{array}{l} \varepsilon_{x}\\ \varepsilon_{y}\\ \gamma_{xy} \end{array}\right\}$$

The off-axis stiffness matrix is as follows:

(9) $$\left[\bar{Q}\right]={\left[T_{\sigma }\right]}^{-1}\left[Q\right]\left[T_{\varepsilon }\right]$$

The relationship of the off-axis stress-strain in the *X*-*Y* coordinate system is as follows:

(10) $$\left\{\begin{array}{l} \sigma_{x}\\ \sigma_{y}\\ \tau_{xy} \end{array}\right\}=\left[\bar{Q}\right]\left\{\begin{array}{l} \varepsilon_{x}\\ \varepsilon_{y}\\ \gamma_{xy} \end{array}\right\}=\left[\begin{array}{ccc} {\bar{Q}}_{11} & {\bar{Q}}_{12} & {\bar{Q}}_{13}\\ {\bar{Q}}_{21} & {\bar{Q}}_{22} & {\bar{Q}}_{23}\\ {\bar{Q}}_{31} & {\bar{Q}}_{32} & {\bar{Q}}_{33} \end{array}\right]\left\{\begin{array}{l} \varepsilon_{x}\\ \varepsilon_{y}\\ \gamma_{xy} \end{array}\right\}$$

### 3.2. Stiffness Analysis of Composite Laminates

**Figure 2:**
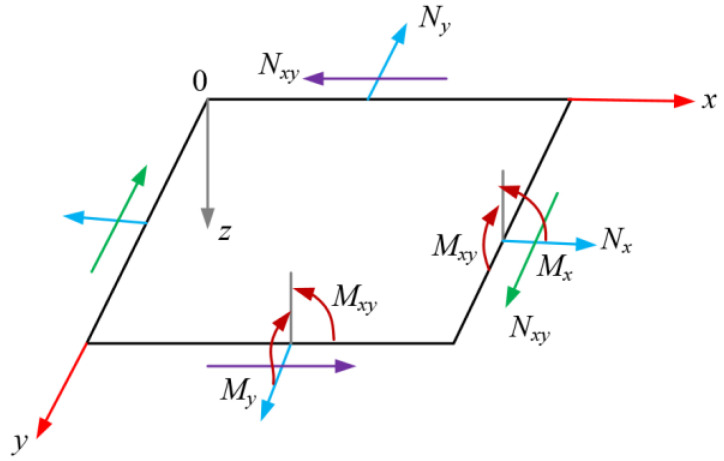
Schematic diagram of the action of plane internal forces and bending moments of the laminate.

The Laminate under the action of plane internal forces and bending moments is shown in Figure 2.

In the figure, *N_x_*, *N_y_*, and *N_xy_* are tensile force, pressure, and shear force per unit length and width of the section, respectively; *M_x_*, *M_y_*, and *M_xy_* are the bending moment and torque per unit length, and width of the section, respectively. They can be obtained by integrating the stress of each single-layer plate along the thickness *t* of the laminate, and the stress-strain relationship is as follows.

(11) $$\left\{\begin{array}{l} N_{x}\\ N_{y}\\ N_{xy} \end{array}\right\}=\int_{-t/2}^{t/2}\left\{\begin{array}{l} \sigma_{x}\\ \sigma_{y}\\ \sigma_{xy} \end{array}\right\}dz,\;\left\{\begin{array}{l} M_{x}\\ M_{y}\\ M_{xy} \end{array}\right\}=\int_{-t/2}^{t/2}\left\{\begin{array}{l} \sigma_{x}\\ \sigma_{y}\\ \sigma_{xy} \end{array}\right\}zdz$$

Equation (11) is the internal force and internal moment in the stress integral form of continuous anisotropic materials. Since the stress distribution along the thickness direction of the laminate is discontinuous, its internal force and moment should be the sum of the internal force and moment of each single-layer.

(12) $$\left\{\begin{array}{l} N_{x}\\ N_{y}\\ N_{xy} \end{array}\right\}=\sum_{k=1}^{n}\int_{t_{k-1}}^{t_{k}}{\left\{\begin{array}{l} \sigma_{x}\\ \sigma_{y}\\ \sigma_{xy} \end{array}\right\}}_{k}dz,\;\left\{\begin{array}{l} M_{x}\\ M_{y}\\ M_{xy} \end{array}\right\}=\sum_{k=1}^{n}\int_{t_{k-1}}^{t_{k}}{\left\{\begin{array}{l} \sigma_{x}\\ \sigma_{y}\\ \sigma_{xy} \end{array}\right\}}_{k}zdz$$

where *t_k_*_−1_ and *t_k_* are the *Z*-directional coordinate values of the upper and lower surfaces of the *k*th layer, respectively.

The strain relationship between internal force and internal moment is as follows:

(13) $$\left\{\begin{array}{l} N_{x}\\ N_{y}\\ N_{xy} \end{array}\right\}={\sum_{k=1}^{n}\left[\begin{array}{ccc} {\bar{Q}}_{11} & {\bar{Q}}_{12} & {\bar{Q}}_{13}\\ {\bar{Q}}_{21} & {\bar{Q}}_{22} & {\bar{Q}}_{23}\\ {\bar{Q}}_{31} & {\bar{Q}}_{32} & {\bar{Q}}_{33} \end{array}\right]}_{k}\times \left\{\left(t_{k}-t_{k-1}\right)\left[\begin{array}{l} \varepsilon_{x}^{0}\\ \varepsilon_{y}^{0}\\ \gamma_{xy} \end{array}\right]+\frac{1}{2}\left(t_{k}^{2}-t_{k-1}^{2}\right)\left[\begin{array}{l} k_{x}\\ k_{y}\\ k_{xy} \end{array}\right]\right\}\;\left\{\begin{array}{l} M_{x}\\ M_{y}\\ M_{xy} \end{array}\right\}={\sum_{k=1}^{n}\left[\begin{array}{ccc} {\bar{Q}}_{11} & {\bar{Q}}_{12} & {\bar{Q}}_{13}\\ {\bar{Q}}_{21} & {\bar{Q}}_{22} & {\bar{Q}}_{23}\\ {\bar{Q}}_{31} & {\bar{Q}}_{32} & {\bar{Q}}_{33} \end{array}\right]}_{k}\times \left\{\frac{1}{2}\left(t_{k}^{2}-t_{k-1}^{2}\right)\left[\begin{array}{l} \varepsilon_{x}^{0}\\ \varepsilon_{y}^{0}\\ \gamma_{xy} \end{array}\right]+\frac{1}{3}\left(t_{k}^{3}-t_{k-1}^{3}\right)\left[\begin{array}{l} k_{x}\\ k_{y}\\ k_{xy} \end{array}\right]\right\}$$

Equation (13) can be converted into:

(14) $$\left\{\begin{array}{l} N_{x}\\ N_{y}\\ N_{xy} \end{array}\right\}=\left[\begin{array}{ccc} A_{11} & A_{12} & A_{13}\\ A_{21} & A_{22} & A_{23}\\ A_{31} & A_{32} & A_{33} \end{array}\right]\left\{\begin{array}{l} \varepsilon_{x}^{0}\\ \varepsilon_{y}^{0}\\ \gamma_{xy} \end{array}\right\}+\left[\begin{array}{ccc} B_{11} & B_{12} & B_{13}\\ B_{21} & B_{22} & B_{23}\\ B_{31} & B_{32} & B_{33} \end{array}\right]\left\{\begin{array}{l} k_{x}\\ k_{y}\\ k_{xy} \end{array}\right\}\;\left\{\begin{array}{l} M_{x}\\ M_{y}\\ M_{xy} \end{array}\right\}=\left[\begin{array}{ccc} B_{11} & B_{12} & B_{13}\\ B_{21} & B_{22} & B_{23}\\ B_{31} & B_{32} & B_{33} \end{array}\right]\left\{\begin{array}{l} \varepsilon_{x}^{0}\\ \varepsilon_{y}^{0}\\ \gamma_{xy} \end{array}\right\}+\left[\begin{array}{ccc} D_{11} & D_{12} & D_{13}\\ D_{21} & D_{22} & D_{23}\\ D_{31} & D_{32} & D_{33} \end{array}\right]\left\{\begin{array}{l} k_{x}\\ k_{y}\\ k_{xy} \end{array}\right\}$$

Define the above coefficient matrices as *A*, *B*, and *D*, where *A* is the in-plane stiffness matrix, *B* is the coupling stiffness matrix, and *D* is the bending stiffness matrix. Each stiffness factor is expressed as follows:

(15) $$\left\{ \begin{array}{l} A_{ij}=\sum_{k=1}^{n}{\left[{\bar{Q}}_{ij}\right]}_{k}\left(z_{k}-z_{k-1}\right)\\ B_{ij}=\frac{1}{2}\sum_{k=1}^{n}{\left[{\bar{Q}}_{ij}\right]}_{k}\left(z_{k}^{2}-z_{k-1}^{2}\right)\\ D_{ij}=\frac{1}{3}\sum_{k=1}^{n}{\left[{\bar{Q}}_{ij}\right]}_{k}\left(z_{k}^{3}-z_{k-1}^{3}\right) \end{array}\right.$$

According to the above equation, the mathematical relationship between the generalized internal force and strain of the laminate is expressed as follows:

(16) $$\left\{\begin{array}{l} N\\ M \end{array}\right\}=\left[\begin{array}{cc} A & B\\ B & D \end{array}\right]\left[\begin{array}{l} \varepsilon_{0}\\ k \end{array}\right]$$

Due to the existence of coupling stiffness matrix *B*, there are not only tension-shear coupling and bending-torsion coupling, but also tension-bending coupling in laminate. In order to prevent in-plane deformation caused by bending internal forces and warpage deformation caused by curing of laminate, a symmetrical layer is used in this article to reduce the coupling effect [45].

### 3.3. Strength Analysis and Failure Criterion of Composite Laminates

Material strength is an important index to measure the load-bearing capacity of a structure, which usually refers to the maximum stress that a material can withstand when it is damaged or experiences failure. The strength indexes of composites include fiber tensile strength, fiber compressive strength, matrix tensile strength, matrix compressive strength, and plane shear strength. The failure criterion is mainly based on the allowable stress and strain of composites, and the failure index of the material is obtained by performing calculations on the layer and matrix. When the failure index is greater than “1”, it indicates that the stress or strain exceeds the allowable range.

Compared with conventional metal materials, composites have more complex strength failure mechanisms. At present, the commonly used theories for evaluating composite damage mainly include maximum stress theory, maximum strain theory, and Hashin failure criterion [46,47]. Although these theories provide methods for evaluating failure modes, they do not address the interaction effects of composites. Tsai and Wu [48] proposed a *Tsai-Wu* strength tensor theory suitable for damage failure of anisotropic materials, which fully considers the inequality and symmetry of each stress component of composites, the tensile and compressive strength of materials, and can predict multiple stress states of composites. Therefore, the Tsai-Wu strength tensor theory is selected as the strength criterion for composite laminates in this article.

In *Tsai-Wu* strength tensor theory, the original failure criterion is summarized as a high-order tensor polynomial criterion, which is generally in the form of:

(17) $$F_{i}\sigma_{i}+F_{ij}\sigma_{i}\sigma_{j}+F_{ijk}\sigma_{i}\sigma_{j}F_{ij}\sigma_{i}\sigma_{k}+\cdots \cdots =1$$

where *σ_i_*, *σ_j_*, *σ_k_* are the stress vectors; *F_i_*, *F_ij_*, *F_ijk_* are the strength coefficients of material properties.

In engineering application, only the first two items are usually taken:

(18) $$F_{i}\sigma_{i}+F_{ij}\sigma_{i}\sigma_{j}=1\left(i,j=1,2,3,4,5,6\right)$$

where *F_i_* is the strength parameter of the material.

For two-dimensional plane stress problem, Equation (18) can be simplified as follows:

(19) $$F_{11}\sigma_{1}^{2}+2F_{12}\sigma_{1}\sigma_{2}+F_{22}\sigma_{2}^{2}+F_{66}\sigma_{6}^{2}+F_{1}\sigma_{1}+F_{2}\sigma_{2}=1$$

The strength parameters in the formula are as follows:

(20) $$\left\{ \begin{array}{l} F_{t}=\frac{1}{X_{t}}-\frac{1}{X_{c}},F_{11}=\frac{1}{X_{t}X_{c}}\\ F_{t}=\frac{1}{Y_{t}}-\frac{1}{Y_{c}},F_{11}=\frac{1}{Y_{t}Y_{c}}\\ F_{66}=\frac{1}{S^{2}},F_{12}=\frac{1}{2\sigma_{0}^{2}}\left[1-\left(\frac{1}{X_{t}}-\frac{1}{X_{c}}+\frac{1}{Y_{t}}-\frac{1}{Y_{c}}\right)\sigma_{0}-\left(\frac{1}{X_{t}X_{c}}+\frac{1}{Y_{t}Y_{c}}\right)\sigma_{0}^{2}\right] \end{array}\right.$$

where *X_t_* is the longitudinal tensile strength; *X_c_* is the longitudinal compressive strength; *Y_t_* is the transverse tensile strength; *Y_c_* is the transverse compression strength; and *S* is the plane shear strength.

The above failure criterion considers that the material fails as long as the maximum stress or strain of the material or a layer exceeds the allowable value of the material. However, it should be noted that in reality, the failure damage of one composite layer does not represent the failure and destruction of other layers, and the material structure still has the bearing capacity. In fact, the layer damage is a progressive damage process, when the stress reaches a certain condition, some components in the composite structure will suffer damage failure, the damage will reduce the load-bearing capacity and stiffness of the damaged area, leading to the redistribution of stress. The stress level on both sides of the damage area increases, and the closer to the damage area, the greater the extent of stress increase. In addition, the more serious the damage to the damage area, the greater the stiffness degradation, and the more serious the stress concentration near the damage area. With the increase in stress or strain, the damage starts to expand, from certain point damage to the layer damage, and then from certain layer damage to other layer damage, and eventually the material stiffness is completely degraded and loses load-bearing capacity.

## 4. Basic Performance Test of Composites

In this article, the CFRP T300/5208 was selected as the raw material for the structural design of composite floor, in which the fiber volume fraction was 60%. In order to obtain the basic performance parameters of the CFRP T300/5208 and provide data support for the subsequent composite floor design, this article conducted 0° and 90° uniaxial tensile tests, 0° and 90° uniaxial compression tests and ±45° in-plane shear tests on the composite [49].

### 4.1. Uniaxial Tensile Tests at 0° and 90° Layer

**Figure 3:**
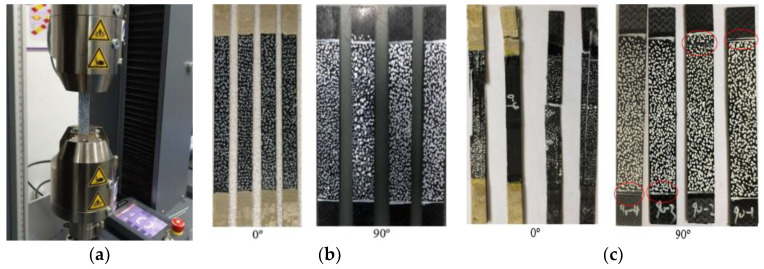
Uniaxial tensile tests at 0° and 90° layer: (**a**) Test machine; (**b**) Uniaxial tensile specimens; (**c**) Failure forms of specimens.

Since excessive bending during uniaxial tensile test of composite materials will cause the specimen to be damaged in advance, even aggravate the dispersion of the basic elastic parameters of the composite and increase the performance test error [50]. Therefore, before the uniaxial tensile test begins, the system alignment was adjusted according to the test requirements to ensure that the bending percentage was controlled in the range of 3–5%, thus reducing the excessive bending caused by the test system. At the same time, in order to ensure that the tensile specimen can be better perpendicular to the upper and lower collets during the test, so that the tensile load direction was always parallel to the longitudinal axis of the specimen, the upper and lower blocks of the system are used for positioning, and the test machine is shown in Figure 3a.

**Figure 4:**
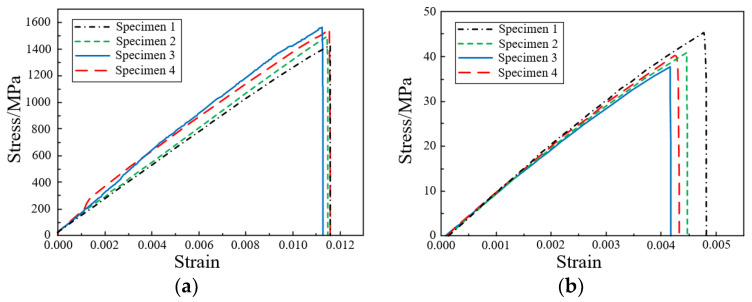
Uniaxial tensile stress-strain curve: (**a**) Specimen at 0° layer; (**b**) Specimen at 90° layer.

The standard specimens at 0° and 90° layer, with dimensions of 250 mm × 15 mm × 1.65 mm and 175 mm × 25 mm × 2.5 mm shall be layered according to [0]_n_ and [90]_n_, respectively. The test specimen is shown in Figure 3b. In order to measure the displacement and strain distribution on the surface of the specimen, the non-contact full-field strain measurement system digital image correlation (DIC) technique was used to measure the specimen. In addition, to eliminate the gap between the specimen and the collet, the specimen was preloaded up to 30% of the failure load and unloaded. Then the tensile performance of the specimens was tested at a constant rate of 2 mm/min until the specimens were damaged. The failure form of the specimen is shown in Figure 3c. The obtained stress-strain curve is shown in Figure 4.

### 4.2. Uniaxial Compression Tests at 0° and 90° Layer

**Figure 5:**
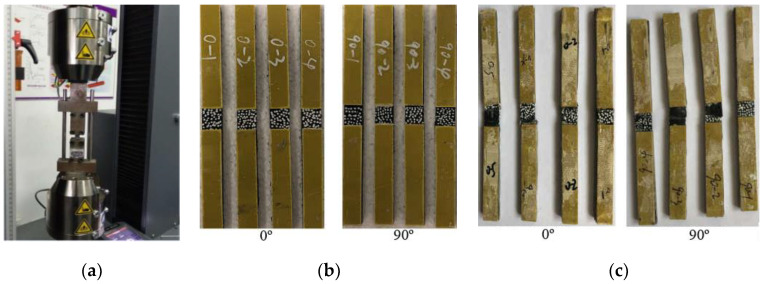
Uniaxial compression tests at 0° and 90° layer: (**a**) Test machine; (**b**) Uniaxial compression specimen; (**c**) Failure forms of specimens.

The uniaxial compression test standard introduced compression force into the specimen through the shear of the contact surface of the wedge chuck, which can effectively avoid the gap problem of the conical wedge block [51]. When the specimen was installed, the end of the specimen can be tightened against the block by tightening the clamping block screws to ensure that the length of the specimen clamped by the upper and lower wedge blocks was equal. In addition, when the specimen was loaded, the stopper ensured that the displacement of the upper and lower wedge was equal, which in turn reduced the bending degree of the specimen and reduced the system error. The adjusted fixture was connected to the center of the test machine platen, where the lower fixture and the lower platen are connected by a ball head to ensure that the compression load direction was always parallel to the longitudinal axis of the specimen. The test machine is shown in Figure 5a.

**Figure 6:**
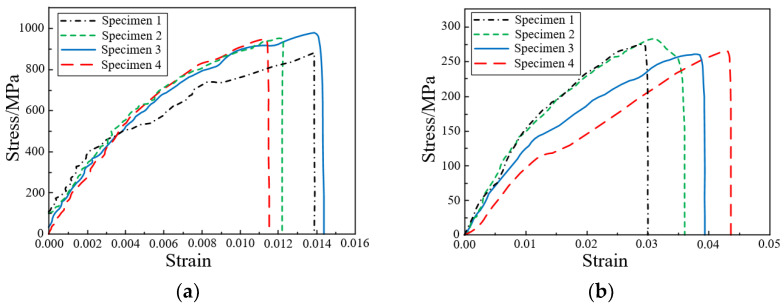
Uniaxial compression stress-strain curve: (**a**) Specimen at 0° layer; (**b**) Specimen at 90° layer.

The standard specimens at 0° and 90° layer, with dimensions of 140 mm × 12 mm × 2.75 mm and 140 mm × 12 mm × 2.9 mm, shall be layered according to [0]_n_ and [90]_n_, respectively. The test specimen is shown in Figure 5b. As the same as the uniaxial tensile test, the displacement and strain distribution on the surface of the specimen are obtained using the non-contact full-field strain measurement system DIC technique. In addition, to eliminate the gap between the specimen and the collet, the specimen was preloaded up to 30% of the failure load and unloaded. Then the compression performance of the specimens was tested at a constant rate of 2 mm/min until the specimens were damaged. The failure form of the specimen is shown in Figure 5c. The obtained stress-strain curve is shown in Figure 6.

### 4.3. In-Plane Shear Test at ±45° Layer

**Figure 7:**
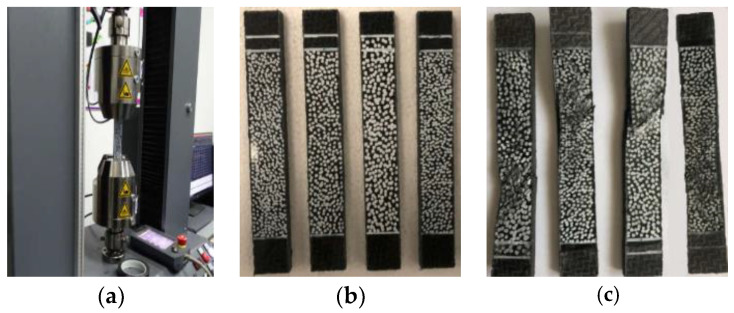
In-plane shear test at ±45° layer: (**a**) Test machine; (**b**) In-plane shear specimen; (**c**) Failure forms of specimens.

**Figure 8:**
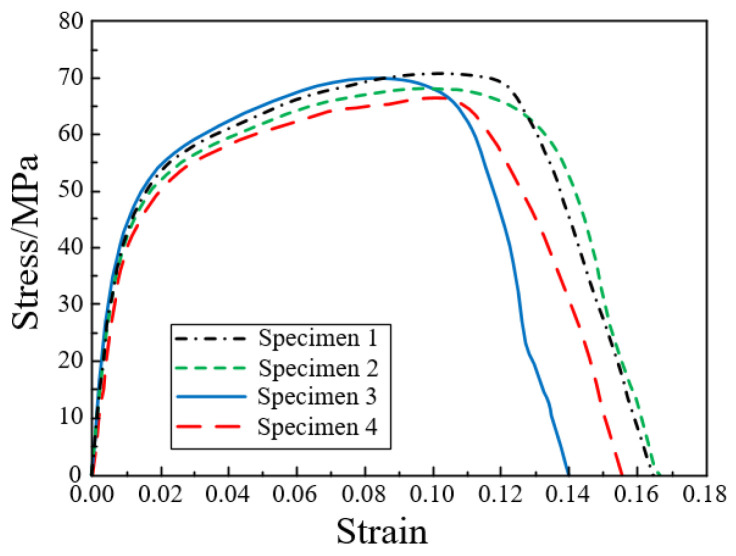
In-plane shear stress-strain curve at ±45° layer.

The ±45° in-plane shear test of composites adopted the same method as the uniaxial tensile test to conduct the uniaxial tensile test on ±45° laminate [52]. The test machine is shown in Figure 7a. The standard specimens at ±45° layer with dimensions of 175 mm × 25 mm × 7 mm, according to the layer of [±45°]_n_. The test specimen is shown in Figure 7b. Similarly, in order to eliminate the gap between the specimen and the collet, the specimen was preloaded up to 30% of the failure load and unloaded. Furthermore, the compression performance of the specimens was tested at a constant rate of 2 mm/min until the specimens were damaged. The failure form of the specimen is shown in Figure 7c. The obtained stress-strain curve is shown in Figure 8.

### 4.4. Performance Test Results of CFRP T300/5208

The basic performance parameters of CFRP T300/5208 obtained through mechanical property tests are shown in Table 3. From the results, it is clear that the CFRP T300/5208 has a small transverse Poisson’s ratio, and when it is subjected to longitudinal tensile and compressive loads, its transverse strain is relatively small, thus more favorable to maintain the transverse mechanical properties of the material.

## 5. Bonding Material Selection and Mechanical Properties Test

In order to meet the connection and assembly requirements of the body metal side panel structure and the CFRP floor, the mechanical properties of the body structure adhesive and the stress characteristics of the bonded joint were tested from the aspects of butt tensile performance and lap shear performance to obtain the tensile and shear parameters of the body structure adhesive.

**Figure 9:**
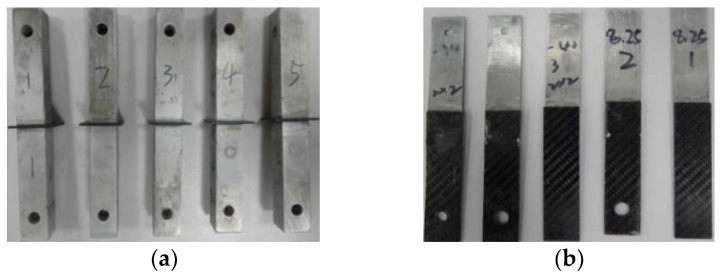
Test specimen: (**a**) Butt tensile specimen; (**b**) Lap shear specimen.

The basic shape of the butt joint specimen is selected as square, the tensile specimen is mainly composed of the upper, middle, and lower parts of bonding substrate and the adhesive layer between them. The upper and lower substrates are DC04 steel with a size of 100 mm × 25 mm × 25 mm, the intermediate base material is CFRP T300/5208, the size is 25 mm × 25 mm × 1.8 mm, and the thickness of the adhesive layer is 0.5 mm [53]. Butt tensile specimen is shown in Figure 9a.

The test steel plate adopts DC04 steel plate with a size is 100 mm × 25 mm × 2.0 mm, and CFRP adopts composites T300/5208 prepreg with a size of 100 mm × 25 mm × 1.8 mm, and the thickness of adhesive layer is 0.5 mm [54]. Lap shear specimen is shown in Figure 9b.

**Figure 10:**
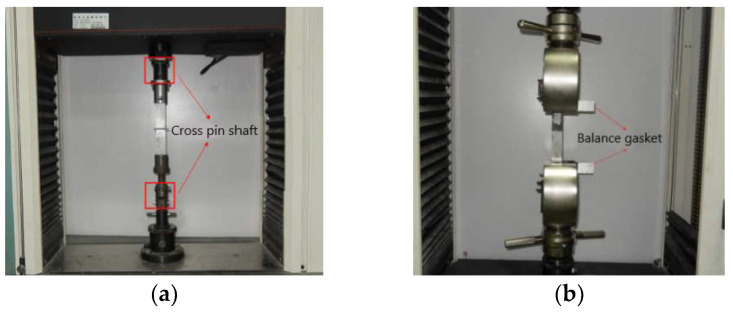
Performance test: (**a**) Butt tensile test; (**b**) Lap shear test.

Araldite 2015 structural adhesive was selected for the assembly connection between CFRP structure and metal material structure, and the butt tensile and single lap shear mechanical properties of Araldite 2015 structural adhesive were investigated, and five sets of tensile and shear tests were conducted, respectively. The butt tensile test and the lap shear test were performed using an electronic universal testing machine, butt tensile and lap shear performance tests are shown in Figure 10.

**Figure 11:**
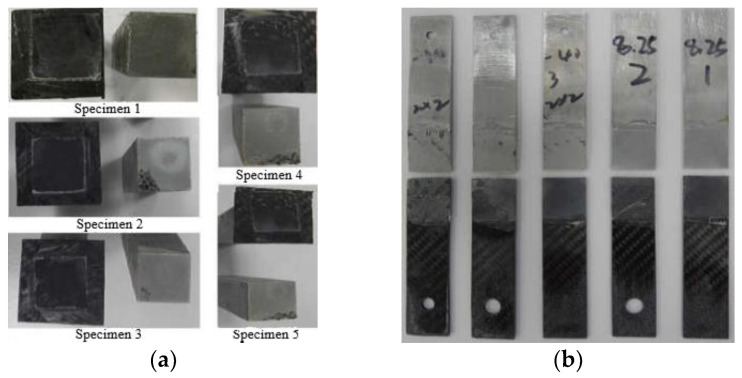
Specimen failure form: (**a**) Butt tensile specimen; (**b**) Lap shear specimen.

**Figure 12:**
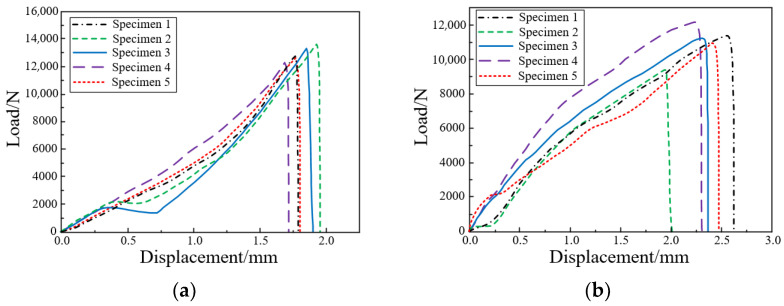
Load-displacement curve: (**a**) Butt tensile specimen; (**b**) Lap shear specimen.

During the test, the specimen was subjected to a tensile test of a loading rate of 2 mm/min until the specimen failed, and the failed specimen and its cross-section is shown in Figure 11. The load-displacement curves of the butt tensile and lap shear specimens obtained by the data acquisition system are shown in Figure 12. The mechanical property parameters of Araldite 2015 structural adhesive obtained through experimental testing and data processing are shown in Table 4.

## 6. Structure and Layer Design of CFRP Floor

### 6.1. Integrated Design of Floor Structure

**Figure 13:**
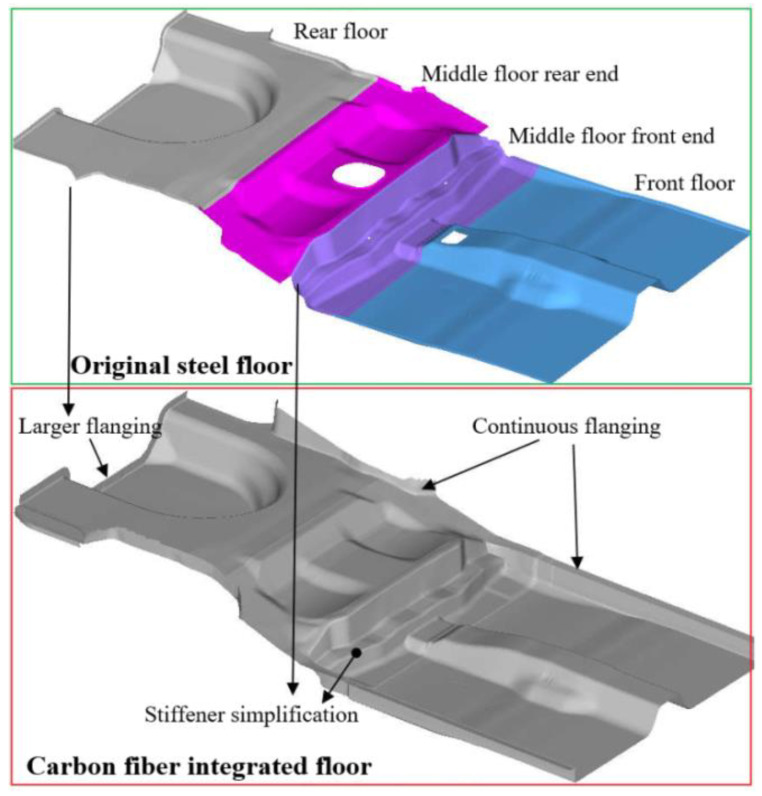
Structure integrated design of the CFRP floor.

Automobile steel floor is mainly composed of front floor, middle floor, and rear floor, with more parts and difficult to integrate molding, while CFRP floor has greater strength and stiffness itself, so some reinforcement ribs can be simplified in structural design and can be integrated design. At the same time, in order to better ensure the continuity of the layer fibers and the continuity of the flow of the resin matrix, the function of structural holes was ignored in the layer design process, and the holes were opened according to the installation position after the sample preparation was completed. In addition, in order to strengthen the connection between the CFRP floor and the metal structure, the connection boundary of the composite floor is expanded to increase the design area of the connection flanging, reduce the connection stress, and increase the connection strength. The structural integration design of the CFRP floor is shown in Figure 13.

### 6.2. Thickness and Block Shape Design of Layer of CFRP Floor

**Figure 14:**
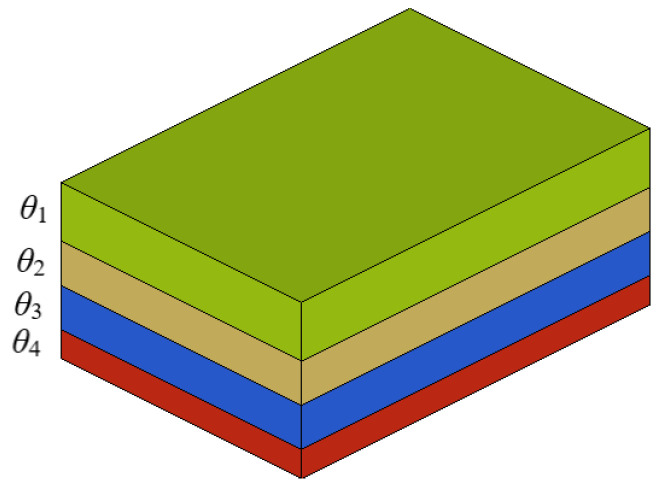
Schematic diagram of super layer.

The CFRP floor mainly exists in the form of laminates, and in the structural layer design stage, each unit grid in the laminate is taken as the basic unit, and the thickness of each layer angle of each unit is adjusted according to the structural performance. To simplify the initial design variables, the layer with the same laying angle is treated as a collection, called a super layer. The super layer is shown in Figure 14, where *θ* indicates the fiber laying angle.

**Figure 15:**
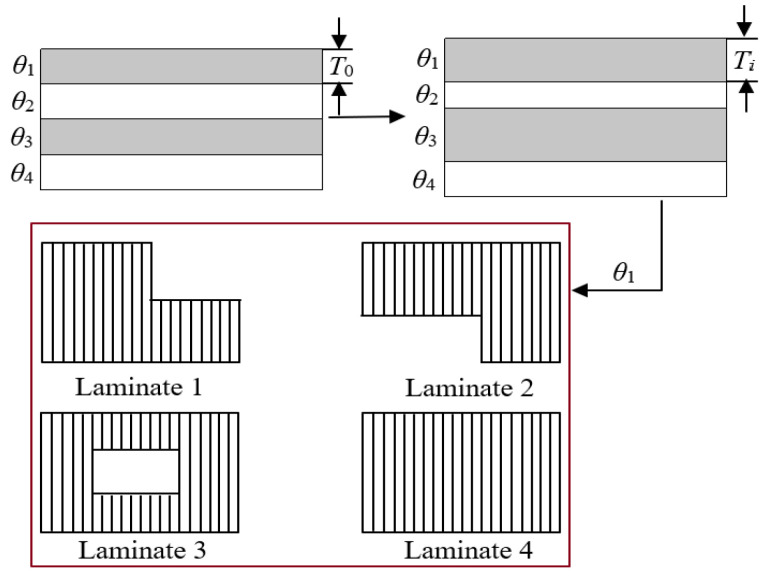
Schematic diagram of super layer analysis.

Since the laying angle of composites is limited by factors such as manufacturing, the laying angles of −45°, 0°, 45°, and 90° in the actual engineering process can not only meet the structural design requirements, but also the laying angle is easy to achieve, which is more conducive to simplifying the production process. Therefore, this article selects the above four commonly used laying angles to carry out the layer design of the automobile floor. Since the super layer is composed of single-layer plate with the same laying angle, in order to determine the shape of each single-layer plates, it is necessary to resolve each super layer into a different shape of laying blocks. The optimal thickness each super layer obtained by the free-size optimization method for each super layer and the different shape layer blocks resolved by each super layer are shown in Figure 15.

In the layer design of CFRP floor structure, the thickness of super layer is taken as the design variable; the lightweight coefficient of body-in-white (BIW) are taken as the optimization objective; the bending stiffness, first-order bending frequency and first-order torsion frequency of the BIW are taken as performance constraints; and also taken is the symmetry of each layer with respect to the neutral plane and the proportion of layers occupied by each laying angle not less than 10% as the manufacturing process constraints; the optimization mathematical model as shown in Equation (21) is constructed.

(21) $$\left\{ \begin{array}{l} find:T=\left(T_{1},T_{2}\cdots \cdots T_{n}\right)\\ min:f\left(T\right)=QLX\\ s.t.:{BS}_{T}\geq {BS}_{0};{BF}_{T}\geq {BF}_{0};{TF}_{T}\geq {TF}_{0};\\ \;\;{CT}_{1};{CT}_{2} \end{array}\right.$$

where *QLX* is the lightweight coefficient; *BS_T_*, *BF_T_* and *TF_T_* are the BIW bending stiffness, first-order bending frequency and first-order torsional frequency, respectively, *BS*_0_, *BF*_0_ and *TF*_0_ are the initial values; *CT*_1_ and *CT*_2_ are the manufacturing process constraints.

**Figure 16:**
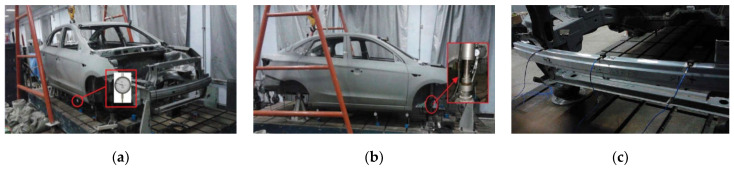
Static stiffness and low-order modal test of BIW: (**a**) Bending stiffness test; (**b**) Torsional stiffness test; (**c**) Low-order modal frequency test.

The static stiffness and low-order modal frequencies of BIW were obtained by tests, and the test conditions are shown in Figure 16, and the test results are shown in Table 5. By comparing the simulation and test values of static stiffness and low-order modal frequency, the error is less than 10%, which meets the accuracy requirements and can be used for optimization design.

**Figure 17:**
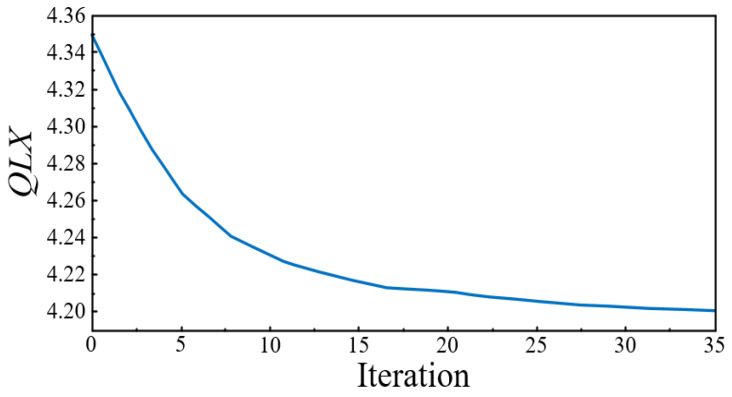
Iterative process of BIW lightweight coefficient.

**Figure 18:**
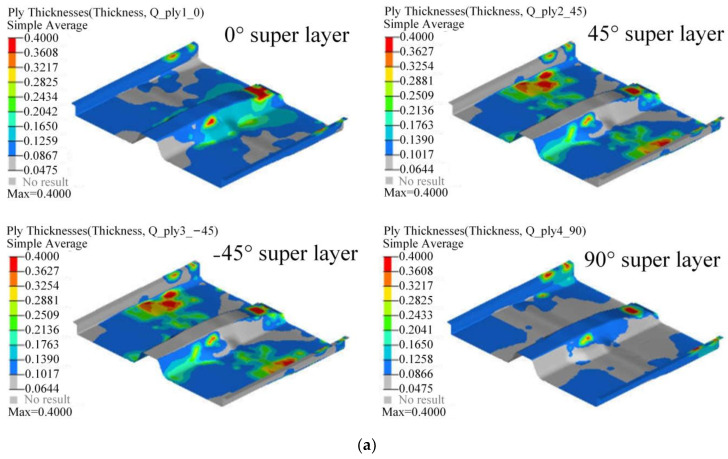

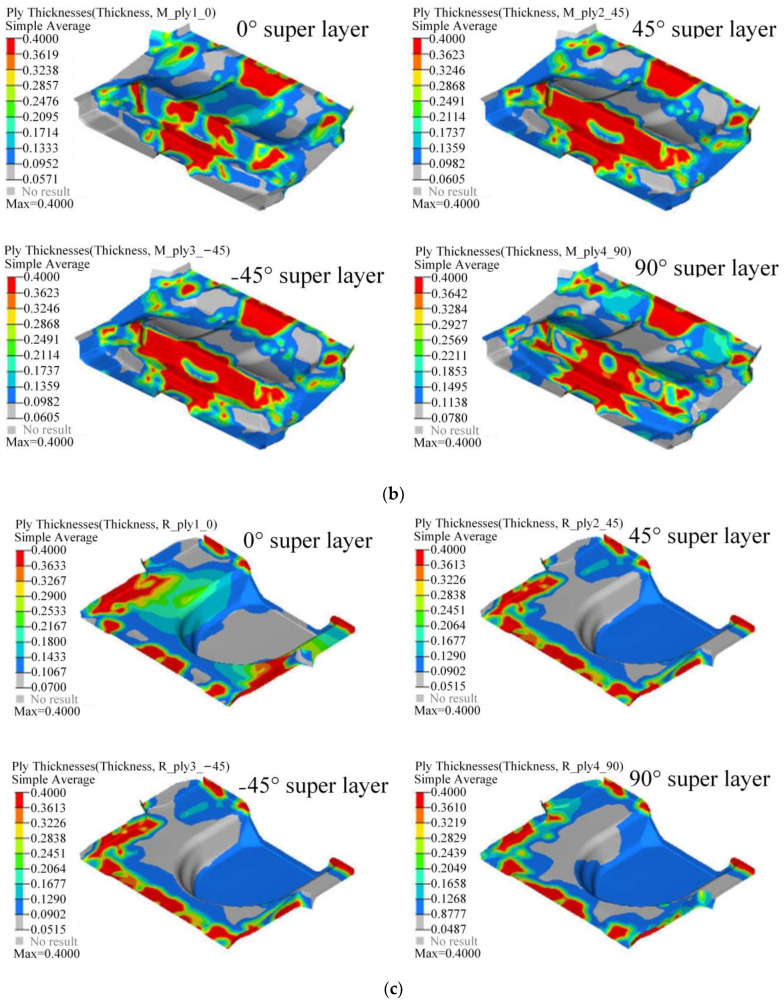
Optimal thickness distribution of super layer: (**a**) Front floor; (**b**) Middle floor; (**c**) Rear floor.

In the optimization process, the initial thickness of each super layer was set to 0.4 mm to ensure sufficient design margin for the super layer. Submitted to Optistruct software for calculation, the lightweight coefficient of the BIW was reduced from 4.35 to 4.20 after 35 iterations. The iterative process of the BIW lightweight coefficient is shown in Figure 17, and the optimal thickness distribution of each super layer obtained by optimization is shown in Figure 18.

**Figure 19:**
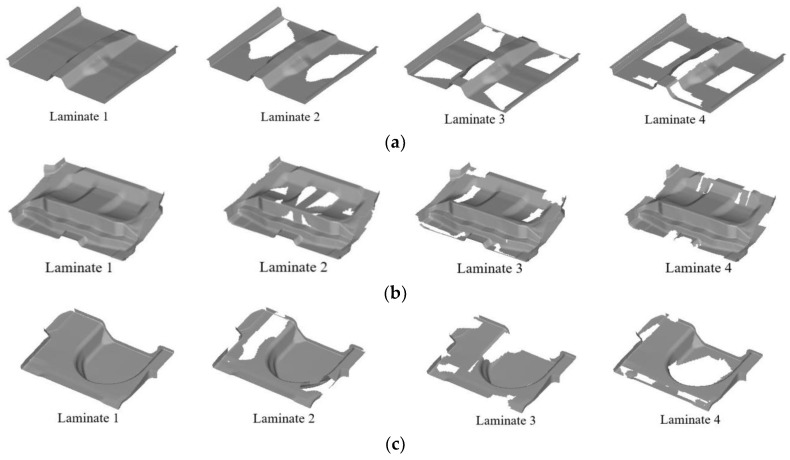
Layer block shape corresponding to 45° super layer: (**a**) Front floor; (**b**) Middle floor; (**c**) Rear floor.

Taking the manufacturing thickness of the laminate as the basic unit, each super layer is resolved into 4 groups of layer blocks of different shapes, and Figure 19 shows the shape of the layer blocks corresponding to a 45° super layer as an example. According to the optimal thickness distribution of the super layer, each floor module corresponds to 4 super layers, so each floor module can resolve 16 different shapes of layer blocks, where the 45° and −45° super layers are restricted by the equilibrium symmetry constraints and have the same layer block shape.

The number range of the parsed layer blocks is defined as 1011–3044, where the first digit of the number represents the numbers of the three design domains of the front, middle, and rear floors, and the corresponding values are 1, 2 and 3; the second and third digits represent the numbers of the 0°, 45°, −45° and 90° super layers, and the corresponding values are 01 to 04; the fourth digit represents the layer blocks shape resolved for each super layer, and the corresponding values are 1 to 4. For example, the number 3024 represents the layer block 4 corresponding to the 45° super layer of the rear floor.

**Figure 20:**
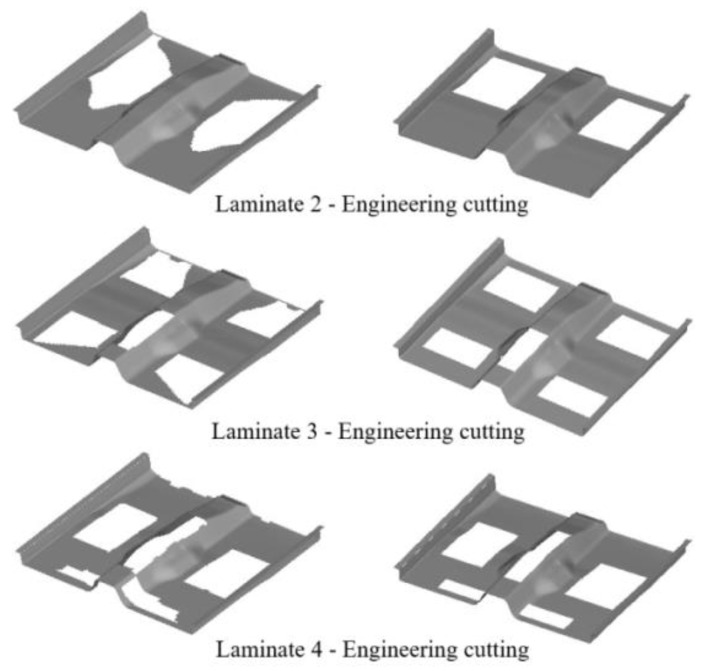
Comparison of the shape of the layer blocks before and after the 45° super layer cut.

The optimized layer blocks are often irregular and not conducive to industrial under-cutting, so the optimized layer blocks need to be regularized to facilitate industrial layer under-cutting. A comparison of the shape of the layer blocks before and after the 45° super layer cut of the front floor module is shown in Figure 20.

### 6.3. Optimizing the Number of Floor Layers

The manufacturable size of single-layer plate commonly used in engineering is 0.125 mm. In order to obtain the specific number of layers of each layer block, the thickness dimension *T_i_* of the layer block is used as the design variable; the floor mass is the optimization objective; the bending stiffness, torsional stiffness and low-order modal frequency of the BIW are used as performance constraints; the *Tsai-Wu* failure criterion is introduced to design the number of half-thickness layers, and the optimized mathematical model constructed is shown in Equation (22).

(22) $$\left\{ \begin{array}{l} find:T_{i}=\left(T_{i1},T_{i2}\cdots \cdots T_{in}\right)\\ min:f\left(T_{i}\right)=M\\ s.t.:BS\left(T_{i}\right)\geq {BS}_{0};TS\left(T_{i}\right)\geq {TS}_{0};\\ \;\;BF\left(T_{i}\right)\geq {BF}_{0};TF\left(T_{i}\right)\geq {TF}_{0};\\ \;\;\mathrm{Tsai-Wu} \end{array}\right.$$

where *M* is the floor mass; *BS*(*T_i_*), *TS*(*T_i_*), *BF*(*T_i_*) and *TF*(*T_i_*) are the BIW bending stiffness, torsional stiffness, first-order bending frequency and first-order torsional frequency, respectively, *BS*_0_, *TS*_0_, *BF*_0_ and *TF*_0_ are the initial values; *Tsai-Wu* is the failure criterion.

The optimal layer thickness *T_i_* of each layer block can be obtained by optimization solution, and the specific layer number *N* of each layer block in the half-thickness layer of CFRP floor is obtained by Equation (23), with a single-layer plate of 0.125 mm thickness as the manufacturing unit.

(23) $$N=T_{i}/0.125$$

where *N* is the number of layers; *T_i_* is the optimal layer thickness.

Due to the influence of the symmetry equilibrium constraint, the floor layer is balanced and symmetrical, so the actual number of layers for each layer block is 2*N*. The half-thickness layer results of the CFRP floor are shown in Table 6. The front floor module has a total of 14.4 × 2 unidirectional layer, the middle floor module has a total of 14.14 × 2 unidirectional layer, and the rear floor module has a total of 12.74 × 2 unidirectional layer.

### 6.4. Structure Layer Modeling of CFRP Floor

**Figure 21:**
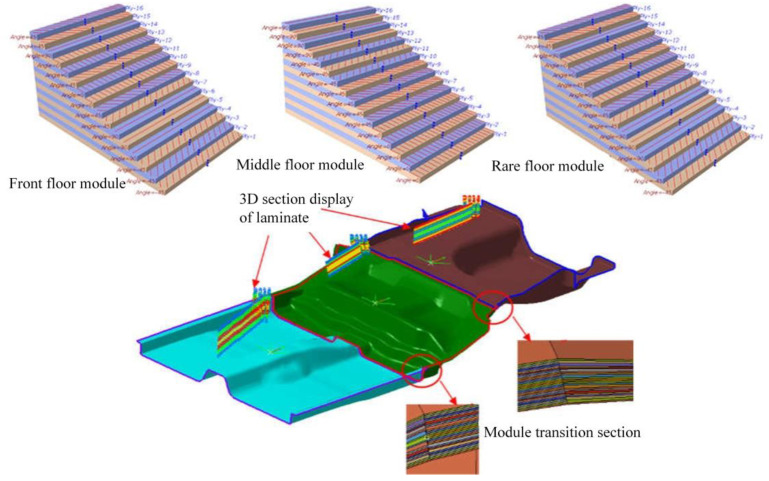
Layer design of CFRP floor.

Using Fibersim 14.0 composite material modeling software, the front, middle, and rear modules of the composite floor are modeled by sub-regional layering with the design method based on regional layering. The specific number of layers of each floor module in Table 6 is rounded to obtain the ply results. The layer design of CFRP floor is shown in Figure 21.

## 7. Performance Verification of CFRP Floor

The floor mass before and after optimization is 24.7 kg and 17.9 kg, respectively. Compared with the original steel floor, the mass of CFRP floor is reduced by 6.8 kg, and the improvement rate is 27.5%. In order to verify the effectiveness of the obtained CFRP floor, the performance of the CFRP floor was verified.

**Figure 22:**
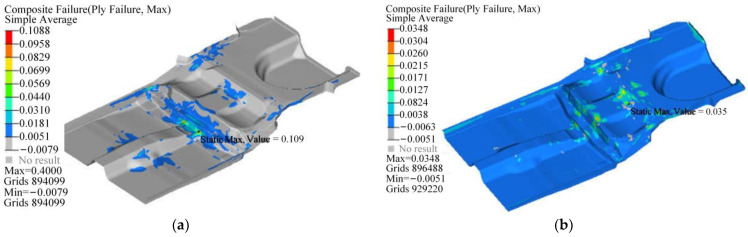
Distribution of failure index of composite floor: (**a**) Bending condition; (**b**) Torsional condition.

**Figure 23:**
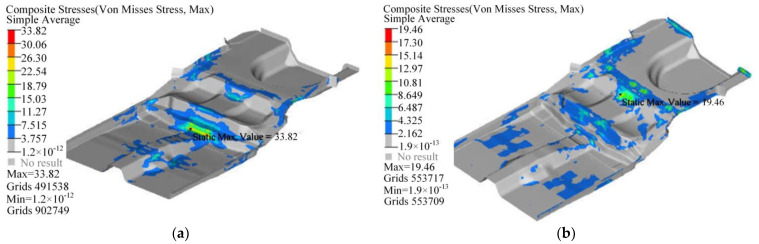
Stress distribution of composite floor: (**a**) Bending condition; (**b**) Torsional condition.

The failure index distribution of the CFRP floor under bending and torsion conditions is shown in Figure 22, and its maximum failure index are 0.109 and 0.035, respectively, which is far less than the failure standard 1. The stress distribution of the CFRP floor under bending and torsion conditions is shown in Figure 23, and the maximum stress are 33.8 MPa and 19.5 MPa, respectively, which are both less than the transverse tensile strength of the composite 40 MPa. Therefore, the designed CFRP floor can better meet the requirements of strength and stiffness while being lightweight and has good fatigue reliability.

## 8. Conclusions

In this article, the basic theory of composite mechanics is expounded from the stress-strain theory of single-layer plates, the stiffness and strength theory of laminate, which provides an important theoretical support for the structural design, material design and allowable value design of composite materials. And the *Tsai-Wu* strength theory is selected as the strength criterion of the CFRP floor laminates. Through the mechanical property tests of CFRP T300/5208 and Araldite 2015 structural adhesive, the basic material parameters were obtained for structural simulation analysis and optimization of the CFRP floor.

The integrated design of the front, middle, and rear floor of the automobile is carried out by using the integrated design characteristics of composites. The shape of the floor super layers is optimized by using the free size optimization method with the BIW lightweight coefficient as the objective and the BIW performance as the constraints. The BIW lightweight coefficient is reduced from 4.35 to 4.20 after free size optimization, and the layer blocks shape are obtained and clipped based on engineering application. With the floor mass as the objective, and the BIW performance as the constraints, the size optimization of the floor layer blocks thickness is optimized. Finally, the number of floor layers are obtained, and the CFRP floor is established in Fibersim software.

A simulation analysis method is then used to compare and verify the performance of the floor before and after optimization. The mass before and after optimization is 24.7 kg and 17.9 kg, respectively. Compared with the original steel floor, the mass of CFRP floor is reduced by 6.8 kg, and the improvement rate is 27.5%. And the failure index of the floor is far less than the failure standard 1. The results show that the design and optimization methods in this article has a significant lightweight effect and integrated manufacturing performance on CFRP floor, while it has a good fatigue reliability.

In this article, our focus is on the study of the layers design and optimization methods of automotive CFRP floor in the continuous variable domain. Furthermore, there is a decimal in the number of layers, as is shown in Table 6, which is not in conformity with the engineering practice, and rounding is also required. However, the number of floor layers cannot be simply rounded, which will affect its mechanical performance and lightweight effect. We plan to propose a series of strategies to solve this problem, which include a rounding strategy for discretization of layers, a domains ply strategy for continuous fiber, and an optimization strategy for layers sequence. However, due to space limitations, these studies will be presented in subsequent research articles.

## Figures and Tables

**Table 1:** Performance parameters of typical reinforced fibers.

Number	Material Parameters	Carbon Fiber T300	E-Glass Fiber	Kevlar 49 Fiber
1	Density [10^3^ kg/m^3^]	1.76	2.54	1.45
2	Tensile strength [GPa]	3.53	1.6	2.9
3	Specific strength [10^5^ m]	2.0	0.63	2.0
4	Tensile modulus [GPa]	230	70	120
5	Specific modulus [10^7^ m]	1.3	0.27	0.83
6	Elongation at break [%]	1.5	3.0	1.9

**Table 2:** Performance parameters of typical thermosetting resins.

Number	Material Parameters	Epoxy Resin	Cyanate Ester Resin	Bismaleimide Resin
1	Bending strength [GPa]	0.0965	0.1619	0.0751
2	Bending modulus [GPa]	3.75	2.89	3.45
3	Bending strain [%]	2.5	8.0	2.2
4	Impact strength [J/m^2^]	21.3	48	16
5	Tensile strength [GPa]	0.0637	0.0868	0.090
6	Tensile modulus [GPa]	2.9	2.89	4.3
7	Elongation at break [%]	2.0	3.8	2.9

**Table 3:** Mechanical property parameters of CFRP T300/5208.

Number	Material Parameters	Value	Number	Material Parameters	Value
1	*X_t_* [GPa]	1.496	8	*S* [GPa]	0.067
2	*X_c_* [GPa]	0.956	9	*G*_12_ [GPa]	6.4
3	*Y_t_* [GPa]	0.040	10	*G*_23_ [GPa]	3.8
4	*Y_c_* [GPa]	0.249	11	*G*_13_ [GPa]	6.4
5	*E*_1_ [GPa]	127.6	12	*µ*_12_	0.28
6	*E*_2_ [GPa]	13	13	*µ*_23_	0.3
7	*E*_3_ [GPa]	10.3	14	*µ*_13_	0.28

**Table 4:** Mechanical properties of Araldite 2015 structural adhesives.

Material Parameters	Tensile Strength	Tensile Modulus	Shear Strength	Shear Modulus
Value	20.5 MPa	1850 MPa	17.8 MPa	502 MPa

**Table 5:** Test results of static stiffness and low-order modal frequency of BIW.

Project	Simulation Value	Test Value	Relative Error
Bending stiffness	12,412.13 N/mm	11,717.12 N/mm	+5.96%
Torsional stiffness	18,643.31 Nm/deg	17,251.95 Nm/deg	+8.06%
First-order bending frequency	52.61 Hz	51.69 Hz	+1.78%
First-order torsional frequency	35.62 Hz	32.53 Hz	+9.50%

**Table 6:** Optimization results of half-thickness layer.

Module	Number	Thickness [mm]	Number of Layers	Number	Thickness [mm]	Number of Layers
Front floor module	1011	0.118	0.94	1031	0.208	1.66
	1012	0.063	0.50	1032	0.113	0.90
	1013	0.084	0.67	1033	0.083	0.66
	1014	0.151	1.21	1034	0.147	1.18
	1021	0.208	1.66	1041	0.096	0.77
	1022	0.113	0.90	1042	0.054	0.43
	1023	0.083	0.66	1043	0.074	0.59
	1024	0.147	1.18	1044	0.062	0.49
Middle floor module	2011	0.102	0.81	2031	0.091	0.72
	2012	0.080	0.64	2032	0.059	0.47
	2013	0.163	1.30	2033	0.14	1.12
	2014	0.207	1.65	2034	0.071	0.56
	2021	0.091	0.72	2041	0.197	1.57
	2022	0.059	0.47	2042	0.130	1.04
	2023	0.140	1.12	2043	0.094	0.75
	2024	0.071	0.56	2044	0.080	0.64
Rear floor module	3011	0.104	0.83	3031	0.209	1.67
	3012	0.068	0.54	3032	0.110	0.88
	3013	0.087	0.69	3033	0.067	0.53
	3014	0.093	0.74	3034	0.046	0.37
	3021	0.209	1.67	3041	0.096	0.77
	3022	0.110	0.88	3042	0.130	1.04
	3023	0.067	0.53	3043	0.091	0.72
	3024	0.046	0.37	3044	0.064	0.51

## Data Availability

Not applicable.
